# Preoperative anemia and transfusion in cardiac surgery: a single-centre retrospective study

**DOI:** 10.1186/s13019-021-01493-z

**Published:** 2021-04-23

**Authors:** Quynh Nguyen, Eric Meng, Joel Berube, Richard Bergstrom, Wing Lam

**Affiliations:** 1 Faculty of Medicine and Dentistry, University of Alberta, Edmonton, Canada; 2 Division of Anesthesiology & Pain Medicine, Department of Medicine, Faculty of Medicine and Dentistry, University of Alberta, Edmonton, Alberta T6G 2S2 Canada

**Keywords:** Pre-operative anemia, Transfusion, Cardiac surgery

## Abstract

**Background:**

Preoperative anemia and transfusion are associated with worse outcomes. This study aims to identify the prevalence of preoperative anemia, transfusion rates on surgery day, and predictors of transfusion in elective cardiac surgery patients at our centre. We also aim to evaluate our preoperative intervention program, and examine the intervention window for anemia before surgery.

**Methods:**

This study included 797 adult patients who underwent elective cardiac surgery at a tertiary hospital. Multivariable logistic regression analysis was used to identify predictors of transfusion on surgery day.

**Results:**

Preoperative anemia was present in 15% of patients. Anemic patients had a significantly higher transfusion rate at 53% compared to 10% in non-anemic patients. Hemoglobin concentration, estimated glomerular filtration rate (eGFR), body surface area (BSA), and total cardiopulmonary bypass time were predictive of transfusion on surgery day. Patients had a median of 7 days between initial visit and surgery day, however, referral to the blood conservation clinic was only done for 8% of anemic patients and treatment was initiated in 3% of anemic patients. Among the 3 anemic patients who received treatment, 2 did not require blood transfusion on surgery day.

**Conclusions:**

Preoperative anemia is present in 15% of patients at our centre and these patients have 53% transfusion rates on surgery day. Hemoglobin concentration, eGFR, BSA, and total cardiopulmonary bypass time were predictors of transfusion on surgery day. Patients had a median of 7 days between initial visit and surgery day. Referral and anemia treatment were infrequently initiated in preoperative anemic patient.

## Background

Anemia is common in patients undergoing surgery, especially in cardiac surgery [1, 2]. The prevalence of preoperative anemia among patients undergoing cardiac surgery is highly variable, depending on the definition of anemia used across studies as well as centre-specific patient populations. Using the World Health Organization (WHO) definition, the general consensus for preoperative anemia prevalence is around 20–30% and increases with age and the presence of comorbidities [2, 3]. The most common causes of preoperative anemia in cardiac surgery patients are hospital-acquired anemia, iron-deficiency anemia, and anemia of chronic disease [4, 5]. The risk of anemia is exacerbated in cardiac surgery patients due to acute hemodilution associated with cardiopulmonary bypass (CPB), perioperative phlebotomy, and surgical blood loss [6]. Preoperative anemia is associated with higher morbidity and mortality, longer intensive care unit (ICU) and hospital stays, and increased health care costs [2, 4, 7, 8].

Preoperative anemia also increases perioperative red blood cell (RBC) transfusion rates in cardiac surgery patients [1, 9]. Allogeneic blood transfusions are a scarce and costly resource that carry with them significant risks including transfusion reactions, transfusion-related immunomodulation, infectious complications, and other systemic adverse effects [1, 9–11]. Similar to preoperative anemia, blood transfusion in cardiac surgery is associated with increased morbidity and mortality, lengths of hospital stay, and health care costs [2, 9]. The relative contribution of preoperative anemia and RBC transfusion to the adverse outcomes in cardiac surgery patients remains uncertain, however, it has been shown that the interaction of the two increases morbidity and mortality far greater than the additive effects of each exposure individually [10, 12–14]. Recognizing the implications of preoperative anemia and perioperative transfusion, interventions to improve the hemodynamic status of patients before surgery have been implemented in multiple centres [15]. These blood conservation programs have been shown to decrease transfusion rates, improve outcomes and reduce associated costs [2, 15]. Preoperative correction of anemia is an essential part of the blood management concept, and is recommended by major professional societies of cardiothoracic surgeons and anaesthesiologists [16–18].

Our study aims to determine the prevalence of anemia, transfusion rates on surgery day, and predictive factors of transfusion in patients undergoing elective cardiac surgery procedures at our institution. We also aim to evaluate our institution’s current preoperative intervention program for anemic patients, and examine the referral window to assess the feasibility of such a program in the cardiac surgery setting.

## Methods

### Patients and study design

The study protocol was reviewed and approved by the Health Research Ethics Board at the University of Alberta, Edmonton, Canada. Due to the retrospective design of the study, individual patient consent was waived. This study included 797 adult (> 18 years of age) patients who had elective cardiac surgery requiring CPB at the Mazankowski Alberta Heart Institute (MAHI), Edmonton, Alberta, Canada from October 2017 to October 2019. Patients were identified from our cardiac pre-admission clinic (PAC) records. If a patient underwent more than one cardiac surgery during the study period, only the initial surgery was included in the study. Emergency procedures and transplantation (heart, lung, heart and lung) were excluded from the study because these procedures do not allow for an accurate assessment of time between PAC visit and surgery day for preoperative intervention purposes.

Patient clinical characteristics, surgical characteristics and outcomes were gathered via review of physical charts scanned and uploaded onto electronic databases maintained by Alberta Health Services. Patient demographics, comorbidities, lab values and imaging parameters were based on the latest available information before PAC visits. The patient and surgical characteristics chosen in this study were based on commonly seen comorbidities in cardiac surgery patients as well as established anemia- and transfusion- associated risk factors. Complex surgical procedures were defined as any combined surgery such as concomitant coronary artery bypass graft (CABG) and valve, double valve, or aortic surgeries.

Following the WHO definition [3], anemia was defined as having a hemoglobin (Hb) concentration of less than 120 g/L in non-pregnant adult females and less than 130 g/L in adult males (Table 1). Further sub-classifications using the WHO criteria were done for mild anemia (Hb concentration between 110 and 120 g/L in females and 110 and 130 g/L in males), moderate anemia (Hb concentration between 80 and 110 g/L in both sexes), and severe anemia (Hb concentration less than 80 g/L in both sexes). Hb values are used as transfusion triggers at our centre; there is no universal Hb threshold for transfusion. Patient conditions and comorbidities are taken into consideration, which accounts for a higher Hb threshold in sicker patients. The general consensus is that otherwise healthy patients with Hb > 100 g/L rarely require perioperative RBC transfusion, and those with Hb < 80 g/L frequently require RBC transfusion. For this study, transfusion on surgery day was defined as any packed RBC (pRBC) transfused in the operating rooms or within 24 h of arrival at the cardiovascular ICU (CVICU).

**Table 1:** Prevalence of anemia among our elective cardiac surgery patients

Anemia degree	Hemoglobin Concentration (g/L)		Prevalence		
	Non-Pregnant Female	Male	Female	Male	Total
Non-Anemic	≥ 120	≥ 130	206	472	678 (85)
Anemia (total)	< 120	< 130	56	63	119 (15)
Mild	110–119	110–129	33	47	80 (67)
Moderate	80–109	80–109	22	16	38 (32)
Severe	< 80	< 80	1	0	1 (1)

Information on patient referral and preoperative treatment for anemia at the local blood conservation clinic was obtained by cross-referencing our patient list with the blood conservation clinic’s patient database. Referral criteria for surgical patients are: having a Hb concentration of 70–130 g/L and a ferritin concentration of less than 100 ng/mL. Upon referral, patients are triaged based on their blood work results and the available amount of time prior to their scheduled surgeries. Patients are contacted within a week and treatments are initiated within 1–2 weeks of their referrals. For treatment, patients are given intravenous iron sucrose, 200 mg infused over a minimum of 15 min × 2 doses, 1 week apart. Unstable cardiac patients are contraindicated for treatment.

### Statistical analysis

Non-parametric continuous variables were expressed as medians with their respective interquartile ranges (IQRs). Categorical variables were expressed as the total number in each category, and the corresponding percentage of the study population they represented. Two-tailed Mann Whitney test and two-sided Fisher’s exact test or chi-square test were used for non-parametric continuous and categorical data, respectively. All candidate variables were used for multivariable logistic regression analysis, with transfusion on surgery day as an outcome. Akaike information criterion (AIC) was used to compare models, and the one with the lowest AIC was selected. Area under the receiver operating characteristic curve (AUC) was used to measure modelling accuracy. Results were reported as odd ratios (ORs) with 95% confidence intervals (CIs). A *p* value of less than 0.05 was considered significant. All statistical analyses were performed using GraphPad Prism version 8.4.3, GraphPad Software, La Jolla California USA.

## Results

### Prevalence of Anemia

Preoperative anemia was present in 119/797 elective cardiac surgery patients (15%) (Table 1). Of these anemic patients, 67% had mild, 32% had moderate, and 1% had severe anemia. Among all anemic patients, 47% were female; in particular, 41% of mildly anemic patients, 58% of moderately anemic patients, and the only severely anemic patient were female.

### Patient characteristics

In terms of demographics, anemic patients were significantly older (*p* = 0.003), more likely to be female (*p* = 0.001), had lower body weight (*p* = 0.002), and smaller body surface area (BSA) (*p* < 0.001) than non-anemic patients (Table 2). Among patients who were anemic, those with moderate/severe anemia were significantly more likely to have lower body weight (*p* = 0.005), lower body mass index (BMI) (*p* = 0.031) and smaller BSA (*p* = 0.006).

**Table 2:** Clinical characteristics of our elective cardiac surgical patients with and without anemia

Parameters	Non-Anemic (***N*** = 678)	Anemic (***N*** = 119)	Total (***N*** = 797)	***P*** value*	Mild Anemic (***N*** = 80)	Mod/Sev. Anemic (***N*** = 39)	***P*** value†
**Demographics**							
Age (yr)	66 (56–73)	69 (63–76)	66 (56–73)	**0.003**	70 (64–76)	66 (57–73)	0.068
Female	206 (30)	56 (47)	262 (33)	**0.001**	33 (41)	23 (59)	0.081
Weight	85 (73–97)	79 (68–90)	83 (71–96)	**0.002**	82 (72–93)	73 (63–84)	**0.005**
BMI (kg/m2)	29 (25–33)	29 (25–33)	29 (25–33)	0.340	29 (25–34)	28 (23–31)	**0.031**
BSA (m2)	2.0 (1.8–2.2)	1.9 (1.7–2.1)	2.0 (1.8–2.1)	**< 0.001**	1.9 (1.8–2.1)	1.8 (1.6–1.9)	**0.006**
**Surgical procedures**			0.175				NA
Isolated CABG	157 (23)	34 (29)	191 (24)		24 (30)	10 (26)	
Isolated valve	290 (43)	55 (46)	345 (43)		35 (44)	20 (51)	
CABG & valve	61 (9)	13 (11)	74 (9)		7 (9)	6 (15)	
Aortic surgery	96 (14)	11 (9)	107 (13)		9 (11)	2 (5)	
Adult congenital	48 (7)	5 (4)	53 (7)		4 (5)	1 (3)	
Others	26 (4)	1 (1)	27 (3)		1 (1)	0 (0)	
**Surgical parameters**							
Repeat sternotomy	74 (11)	15 (13)	89 (11)	0.635	13 (16)	2 (5)	0.139
Cross-clamp time (min)	84 (61–110)	77 (59–103)	83 (61–110)	0.252	80 (58–106)	76 (59–102)	0.777
Total bypass time (min)	106 (80–141)	99 (81–136)	104 (80–141)	0.455	102 (80–144)	95 (84–127)	0.551
DHCA	32 (5)	6 (5)	38 (5)	0.817	6 (8)	0 (0)	0.176
**Co-morbidities**							
Diabetes	161 (24)	58 (49)	219 (27)	**< 0.0001**	38 (48)	20 (51)	0.845
CVA	41 (6)	14 (12)	55 (7)	**0.030**	6 (8)	8 (21)	0.065
**Lab and imaging parameters**							
eGFR (ml/min/1.73 m2)	80 (66–92)	60 (41–85)	78 (63–92)	**< 0.0001**	67 (45–85)	56 (18–83)	**0.046**
LVEF (%)				**0.029**			**0.004**
≥ 50	560 (83)	92 (77)	652 (82)		66 (83)	26 (67)	
< 50	95 (14)	27 (23)	122 (15)		14 (18)	13 (33)	
**Preoperative intervention**							
Referral to BCC	NA	9 (8)	NA	NA	5 (6)	4 (10)	0.473
Received treatment at BCC	NA	3 (3)	NA	NA	0 (0)	3 (8)	**0.033**
Days from PAC to surgery	7 (2–15)	7 (2–17)	7 (2–15)	0.990	7 (1–14)	7 (2–18)	0.537

*BCC* blood conservation clinic, *BMI* body mass index, *BSA* body surface area, *CABG* coronary artery bypass graft, *CVA* cerebrovascular accident, *DHCA* deep hypothermic circulatory arrest, *eGFR* estimated glomerular filtration rate, *Hb* hemoglobin, *LVEF* left ventricular ejection fraction, *min* minute, *mod/sev* moderate/severe, *PAC* pre-admission clinic, *P* value * *p* value between anemic and non-anemic groups, *P* value† *p* value between mild anemic and moderate/severe anemic groups, *yr* year

There were no differences in the surgical procedures performed between anemic and non-anemic patients, as well as between patients with different degrees of anemia. The majority of patients underwent isolated CABG or isolated valve surgery, followed by aortic surgery, combined CABG and valve, adult congenital and other surgeries. Both patient groups also had similar frequencies of having a repeat sternotomy or undergoing deep hypothermic circulatory arrest (DHCA) during their surgeries. The cross-clamp time and total bypass time were not significantly different among groups.

Anemic patients were significantly more likely to have diabetes (*p* < 0.0001) or a history of cerebrovascular accident (CVA) (*p* = 0.030). There were no differences regarding these co-morbidities between mild and moderate/severe anemic patients. Laboratory and imaging data showed that anemic patients had significantly lower estimated glomerular filtration rate (eGFR) (*p* < 0.0001) and a higher proportion of these patients had reduced left ventricular ejection fraction (LVEF, LVEF< 50%) (*p* = 0.029). Compared to mild anemic patients, moderate/severe anemic patients were more likely to have lower eGFR (*p* = 0.046) and reduced LVEF (*p* = 0.004) values.

### Transfusion rates and outcomes in elective cardiac surgery patients

Anemic patients had a significantly higher transfusion rate at 53% compared to 10% in non-anemic patients (*p* < 0.0001) (Table 3). Among patients with anemia, 79% of moderate/severe anemic patients and 40% of mild anemic patient required transfusion (*p* < 0.0001). There were no differences in terms of number of pRBCs transfused across groups. The majority of patients received 1–2 pRBCs on surgery day.

**Table 3:** Transfusion rates and outcomes among elective cardiac surgery patients

Transfusion	Non-Anemic (***N*** = 678)	Anemic (***N*** = 119)	***P*** value*	Mild Anemic (***N*** = 80)	Moderate/Severe Anemic (***N*** = 39)	***P*** value†
**Transfusion rate**	65 (10)	63 (53)	**< 0.0001**	32 (40)	31 (79)	**< 0.0001**
**pRBC transfused**			0.915			NA
1	26 (40)	26 (41)		13 (41)	13 (42)	
2	20 (31)	17 (27)		8 (25)	9 (29)	
3	6 (9)	4 (6)		0 (0)	4 (13)	
4	6 (9)	8 (13)		6 (19)	2 (6)	
≥ 5	7 (11)	8 (13)		5 (16)	3 (10)	
**Outcomes**						
Length of CVICU stay (d)	2 (1–4)	3 (1–6)	**0.001**	3 (1–6)	3 (2–6)	0.611
Length of hospital stay (d)	7 (5–9)	10 (6–16)	**< 0.0001**	9 (6–16)	10 (7–14)	0.319
In-hospital mortality	8 (1)	11 (9)	**< 0.0001**	6 (8)	5 (13)	0.501

*CVICU* cardiovascular intensive care unit, *d* day, *pRBC* packed red blood cells, *P* value * *p* value between anemic and non-anemic groups, *P* value† *p* value between mild anemic and moderate/severe anemic groups

With regards to outcomes, anemic patients had significantly worse outcomes as indicated by longer CVICU stays (*p* = 0.001), longer hospital stays (*p* < 0.0001), and higher incidences of in-hospital mortality (p < 0.0001) compared to non-anemic patients. Patients with preoperative anemia spent a median of 3 days in the CVICU and a median of 10 days in the hospital. The in-hospital mortality rate was 9% in anemic patients compared to 1% in non-anemic patients. These outcomes were similar between anemic patients of varying severity.

### Predictive factors of transfusion on surgery day in elective cardiac surgery patients

Multivariate logistic regression analysis of all 797 patients showed that Hb concentration (OR = 0.930, 95% CI = 0.912–0.947), eGFR (OR = 0.979, 95% CI = 0.966–0.992), BSA (OR = 0.018, 95% CI = 0.005–0.067) and total CPB time (OR = 1.013, 95% CI = 1.008–1.019) were predictive of transfusion on surgery day in elective cardiac surgery patients (Table 4). The AUC was 0.8886.

**Table 4:** Predictive factors of same day transfusion using multivariable logistic regression analysis for patients undergoing elective cardiac surgery

Parameters	Odds Ratio	95% CI		***P*** value
		Lower	Upper	
**Demographics**				
Age	1.011	0.990	1.034	0.308
Sex	0.870	0.465	1.607	0.658
BSA	0.018	0.005	0.067	**< 0.0001**
**Surgical parameters**				
Complex surgical procedures	1.737	0.886	3.375	0.105
Repeat sternotomy	1.575	0.689	3.518	0.273
Total bypass time	1.013	1.008	1.019	**< 0.0001**
DHCA	1.022	0.355	2.841	0.967
**Co-morbidities**				
Diabetes	1.168	0.650	2.072	0.598
CVA	1.456	0.625	3.240	0.369
**Lab and imaging parameters**				
eGFR	0.979	0.966	0.992	**0.002**
Reduced LVEF	1.034	0.511	2.023	0.925
Hemoglobin	0.930	0.912	0.947	**< 0.0001**

*BSA* body surface area, *CI* confidence interval, *CVA* cerebrovascular accident, *DHCA* deep hypothermic circulatory arrest, *eGFR* estimated glomerular filtration rate, *LVEF* left ventricular ejection fraction

### Current preoperative intervention and referral windows

**Fig. 1:**
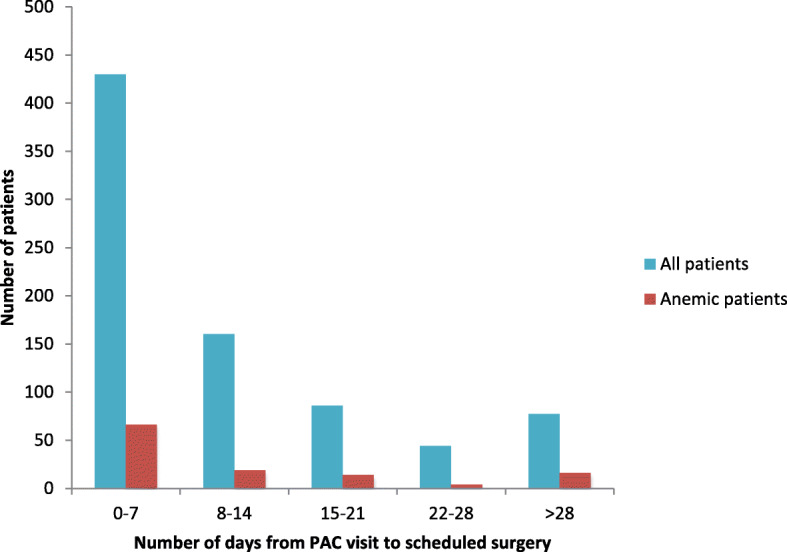
Number of days from pre-admission clinic visit to scheduled surgery day in all patients (*N* = 797) and anemic patients (*N* = 119). PAC, pre-admission clinic

Among anemic patients, 9 patients (8%) were referred to the blood conservation clinic and 3 patients (3%) received treatment with iron therapy (Table 2). Among the 9 patients with referrals, 5 were in the mild anemia category and 4 were in the moderate/severe anemia category. All 3 anemic patients who received treatment were from the moderate/severe group; 2 did not require blood transfusion and one received 1 pRBC on surgery day. The most common reasons for not receiving treatment in those who were referred include inadequate time between referral and surgery date, not meeting criteria for treatment, and patient no-shows or appointment cancellations. There were no differences in the frequencies of referral to the blood conservation clinic between patients with different degrees of anemia. Once the referral has been made, only moderate/severe anemic patients were treated in our cohort (*p* = 0.033). In terms of referral windows, the median time from PAC visit to surgery date was 7 days, and there were no significant differences across all groups. The majority of patients (430/797 total patients, 54%; 66/119 anemic patients, 55%) had 7 days or less between their PAC visits and surgery dates (Fig. 1).

## Discussion

We found that the prevalence of preoperative anemia in elective cardiac surgery patients at our centre is 15%, and that the majority of anemic patients were classified as having mild or moderate anemia according to the WHO definition. Using an Hb concentration less than 125 g/L as the definition of anemia, a multicentre study found the overall prevalence of preoperative anemia in their cardiac surgery patients to be 26%, with values ranging from 22% to 30% at participating hospitals [4]. Another multicentre study identified the prevalence of anemia among cardiac surgery patients to be 31%, with centre-specific prevalence varying from 23% to 45% [7]. Other studies revealed differing prevalences of anemia in preoperative cardiac surgery patients, ranging from 15% to over 54% depending on the definition of anemia used in each study as well as specific patient population characteristics [2, 6, 8, 19]. Since we excluded all emergency and transplant cases from our study, this could explain the lower prevalence of anemia in preoperative cardiac surgery patients at our centre compared to other studies. However, the variation in anemia prevalence across different studies is apparent from the literature, and the exact cause for this is unknown [2, 4, 7]. Further studies are required to elucidate the causes of this variation, as they will be important to guide management as well as preoperative blood conservation strategies.

In terms of transfusion rates, 53% of our anemic patients and 10% of our non-anemic patients required pRBC transfusion on the day of surgery. The need for blood transfusion was significantly higher in moderate/severe anemic patients (79%) compared to mild anemic patients (40%). These findings are consistent with previous studies at other centres. Klein et al. found a 64% transfusion rate in anemic and a 37% transfusion rate in non-anemic patients across multiple centres [7]. A single centre observational study reported RBC transfusion in 50% of anemic and 15% of non-anemic cardiac surgery patients [13]. A previous systematic review also revealed more anemic patients receiving RBC transfusion than non-anemic patients (33% vs 12%, respectively) [8]. Anemia has been established as a strong predictor of perioperative transfusion in cardiac surgery patients [2, 20–22]. Our study supports this observation and highlights the need for blood conservation in this patient population. In addition, we found no differences in the number of pRBC units transfused between anemic and non-anemic patients who required blood transfusion. A previous study, however, showed that patients with anemia received more units of RBCs compared to those without anemia [7]. The differences in sample sizes, patient populations and transfusion practices across different centres may explain these findings.

From our model, Hb concentration, eGFR, BSA, and total bypass time were found to be associated with transfusion on surgery day in cardiac surgery patients. Anemia is a known predictor of transfusion during surgery and the relationship has been well established in previous studies. The positive correlation between CPB time and risk of allogeneic blood transfusion has also been described in the literature [22–24]. Longer CPB time may lead to more inflammation, impaired hemostasis, increased fibrinolysis, and consequently increased blood loss and transfusion requirements. In addition, kidney function and BSA have been shown to be predictive of transfusion in cardiac surgery and both have been included in multiple risk score models to predict transfusion in cardiac surgery [25–28]. These findings support the validity of our model.

One of the goals of this study was to look into our preoperative intervention for anemic patients and identify any potential areas for improvement. Among anemic patients, 8% were referred to the blood conservation clinic and 3% received treatment with iron therapy. Treatments were only initiated in moderate/severe anemic patients upon referral. Our elective cardiac surgery patients had a median of 7 days between their PAC visit and operation day, with the majority of patients having 7 days or less. Although patients were triaged based on their anemic status and available time for treatment prior to surgery, a typical course of treatment with iron therapy at our centre is at least 1 week, not taking into account referral, pre-treatment consultation, and treatment planning. This short window for preoperative correction of anemia explains why only a small percentage of our anemic patients got treated before their scheduled surgeries. Even though our cohort comprises of elective patients, cardiac surgery is often performed in a semi-acute setting where delaying surgery for preoperative anemia correction may carry more risk than benefit. The balance between anemia and transfusion risks versus the risks of delaying surgery needs to be taken into consideration, and evaluated on a case-to-case basis. It would be interesting to compare and contrast the time between initial visit and surgery day at other centres, as well as the effectiveness of their blood management program for elective cardiac surgery patients. In addition, patient referral to preoperative anemia correction was infrequently done, however, the reasons behind that remain to be determined.

Various preoperative blood management strategies have been studied such as iron therapy (oral and intravenous), erythropoietin administration, dietary supplements of vitamin B12 and folate, or preoperative autologous blood donation [15, 29, 30]. A recent single-centre randomised controlled study found that a combination treatment of intravenous iron, subcutaneous erythropoietin alpha, vitamin B12, and oral folic acid on the day before surgery reduced RBC and total allogeneic blood product transfusions in patients with preoperative anemia or isolated iron deficiency undergoing elective cardiac surgery [31]. This is particularly relevant since it makes it possible to intervene in patients whose elective cardiac surgery is scheduled within a few days after an acute cardiac event.

The limitations of this study include all those of the single centre retrospective design. Our anemic patient sample size is also small, which may limit the generalizability of our findings. Health care provider preferences may affect the decision of whether or not to transfuse, as well as of how many pRBC units will be transfused in cases when a transfusion is decided. In addition, referral of anemic patients to the blood conservation clinic prior to surgery was not consistently done at our centre. The small number of anemic patients who got referred and treated made it difficult to assess the effectiveness of the blood conservation program at our centre. Other factors may influence the transfusion rates as well, but were not included in our study.

## Conclusions

Preoperative anemia, defined using the WHO criteria, was present in 15% of our elective cardiac surgery patients with the majority of them classified as being mild or moderately anemic. Transfusion rate during surgery day was significantly higher at 53% in preoperative anemic patients compared to 10% in non-anemic patients. Hb concentration, eGFR, BSA, and total bypass time were found to be predictive of transfusion on surgery day among our elective cardiac surgery patients. Our patients had a median of 7 days between initial visit and surgery day. Referral to the blood management program and initiation of treatment were seldom done for anemic patients. A shift in the preoperative intervention pathway is needed at our centre to adequately correct anemia in anemic patients prior to surgery; however, this requires careful weighing of the risks of anemia and transfusion versus the risk of delaying surgery.

## Data Availability

The datasets used and/or analysed during the current study are available from the corresponding author on reasonable request.

## Abbreviations

AIC: Akaike information criterion
AUC: Area under the receiving operating characteristic curve
BMI: Body mass index
BSA: Body surface area
CABG: Coronary artery bypass graft
CBP: Cardiopulmonary bypass
CIs: Confidence intervals
CVA: Cerebrovascular accident
CVICU: Cardiovascular intensive care unit
DHCA: Deep hypothermic circulatory arrest
eGFR: estimated glomerular filtration rate
Hb: Hemoglobin
ICU: Intensive care unit
IQRs: Interquartile ranges
LVEF: Left ventricular ejection fraction
ORs: Odd ratios
PAC: Pre-admission clinic
pRBC: packed red blood cells
RBC: Red blood cell
WHO: World Health Organization
